# What do end-users want to know about managing the performance of healthcare delivery systems? Co-designing a context-specific and practice-relevant research agenda

**DOI:** 10.1186/s12961-021-00779-x

**Published:** 2021-10-11

**Authors:** Jenna M. Evans, Julie E. Gilbert, Jasmine Bacola, Victoria Hagens, Vicky Simanovski, Philip Holm, Rebecca Harvey, Peter G. Blake, Garth Matheson

**Affiliations:** 1 DeGroote School of Business, McMaster University, 1280 Main Street West, Hamilton, ON L8S4M4 Canada; 2 Institute of Health Policy, Management, and Evaluation, University of Toronto, Toronto, ON Canada; 3 Ontario Health (Cancer Care Ontario), Toronto, ON Canada; 4 Ontario Health (Ontario Renal Network), Toronto, ON Canada; 5 London Health Sciences Centre, London, ON Canada

**Keywords:** Research priority-setting, Research co-design, Performance management, Performance incentives, Performance feedback, Behaviour change, Embedded research, Healthcare, Network governance

## Abstract

**Background:**

Despite increasing interest in joint research priority-setting, few studies engage end-user groups in setting research priorities at the intersection of the healthcare and management disciplines. With health systems increasingly establishing performance management programmes to account for and incentivize performance, it is important to conduct research that is actionable by the end-users involved with or impacted by these programmes. The aim of this study was to co-design a research agenda on healthcare performance management with and for end-users in a specific jurisdictional and policy context.

**Methods:**

We undertook a rapid review of the literature on healthcare performance management (*n* = 115) and conducted end-user interviews (*n* = 156) that included a quantitative ranking exercise to prioritize five directions for future research. The quantitative rankings were analysed using four methods: mean, median, frequency ranked first or second, and frequency ranked fifth. The interview transcripts were coded inductively and analysed thematically to identify common patterns across participant responses.

**Results:**

Seventy-three individual and group interviews were conducted with 156 end-users representing diverse end-user groups, including administrators, clinicians and patients, among others. End-user groups prioritized different research directions based on their experiences and information needs. Despite this variation, the research direction on motivating performance improvement had the highest overall mean ranking and was most often ranked first or second and least often ranked fifth. The research direction was modified based on end-user feedback to include an explicit behaviour change lens and stronger consideration for the influence of context.

**Conclusions:**

Joint research priority-setting resulted in a practice-driven research agenda capable of generating results to inform policy and management practice in healthcare as well as contribute to the literature. The results suggest that end-users are keen to open the “black box” of performance management to explore more nuanced questions beyond “does performance management work?” End-users want to know how, when and why performance management contributes to behaviour change (or fails to) among front-line care providers.

**Supplementary Information:**

The online version contains supplementary material available at 10.1186/s12961-021-00779-x.

## Introduction

Research agendas are typically driven by investigator interests, resulting in studies that may fail to address questions of relevance to end-users or convey information aligned with end-user needs [1]. An end-user is an individual, community or organization outside of academia that will use or benefit from the results of research [2]. Involvement of end-user groups in setting research agendas is critical for three reasons [3]. First, in terms of ethics, individuals most affected by particular phenomena should have a say in shaping the direction of associated research. Second, in terms of impact, when research reflects the needs and interests of end-users, the results are more likely to be used. Third, in terms of efficiency, considerable resources are wasted when applied research fails to contribute to practice or policy due to irrelevance or triviality of the topic [4, 5]. Joint identification of research priorities enhances the likelihood of conducting research that matters to and can be applied by end-users.

In the healthcare field, involving end-users in research priority-setting is increasingly recognized as a best practice [6, 7]. The lived experiences of end-users, such as patients and clinicians, can enhance the quality and relevance of research [8] and address criticism that most clinical research is “nonuseful” [9]. Many healthcare studies have found dramatic mismatches in research priorities between researchers, patients and clinicians [10, 11]. For example, there is a preoccupation with studying pharmaceutical interventions rather than education and nondrug therapies that patients and clinicians prefer as treatment options [10, 11]. To address research–practice gaps such as these, joint priority-setting is increasingly common. A notable example is the James Lind Alliance (JLA), an initiative that brings together clinicians, patients and caregivers in priority-setting partnerships (PSPs) to jointly identify research priorities for specific conditions or healthcare settings [12, 13]. Over 125 PSPs have been conducted since 2007 following the JLA method in the United Kingdom, Canada and other countries [14].

The research–practice gaps described above are not unique to the field of healthcare. In the management discipline, scholars have criticized management studies for “benefit[ing] no one but the authors” [15], for being “pointless” [16] and for being theory-driven rather than problem-driven and phenomenon-based [17]. Practising managers contend that management research is “trivial”, “incomprehensible” and “irrelevant” to their day-to-day challenges [18, 19]. Management studies have shown considerable differences in research priorities between researchers and end-users [20–23]. In response, management scholars have been calling for more end-user engagement through “partnered research” [24], “evidence co-creation” [25], “research collaboration with allies” [26] and “participatory organizational research” [27] in which researchers and end-users collaborate throughout the research process, including in the generation of research questions.

Despite increasing interest in joint research priority-setting in both the healthcare and management disciplines, few studies at the intersection of healthcare and management engage end-user groups in setting research priorities. Drawing from organization and management science, sociology and organizational psychology, healthcare management research examines the impact of management and organizational practices on performance in the healthcare sector. Given increasing attention to quality deficits in health systems and calls for more accountability for care [28, 29], involving end-user groups in setting research agendas for healthcare management research is increasingly important.

Prominent among efforts to improve and account for care have been large-scale policies and programmes aimed at incentivizing performance. For example, in the Merit-Based Incentive Payment System in the United States, clinicians receive a payment bonus, payment penalty or no payment adjustment based on their performance in four areas: quality, improvement activities, promoting interoperability and cost [30]. Similarly, in Ontario, Canada, hospitals are mandated under the Excellent Care for All Act to develop annual quality improvement plans and to tie executive compensation to meeting the performance targets established in those plans [31]. “Performance management” (PM) programmes like these provide performance feedback and establish accountability for performance outcomes using managerial tools such as contracts, targets, scorecards and incentives with the aim of influencing behaviour and results [32, 33]. We use the term “performance management” rather than “performance measurement” because we are interested in the mechanisms used to stimulate action in response to performance feedback, rather than in indicator development and selection. While much research has been conducted on healthcare PM, the evidence base is mixed, and there are reports of unintended negative consequences [34–45]. The state of the literature signals a need for research that answers more nuanced questions about PM. Involving end-user groups in setting a research agenda for PM may generate results that are more meaningful and actionable. To our knowledge, there are no examples of joint research priority-setting with end-users who are involved with or impacted by PM programmes.

The aim of this study was to co-design a research agenda on PM of healthcare delivery systems with and for end-users in a specific jurisdictional and policy context. A government agency that oversees the performance of 40 service delivery networks using a suite of PM tools and processes hired an embedded researcher to lead this work. The researcher collaborated closely with end-users inside the organization, undertook a rapid review of the literature and consulted with diverse end-user groups (within and outside of the organization) via semi-structured interviews that included quantitative ranking to prioritize directions for future research on PM. Joint priority-setting resulted in a practice-driven research agenda capable of generating results to inform management practice as well as contribute to the literature. We use the Reporting Guideline for Priority-Setting of Health Research (REPRISE) to comprehensively describe this study in the pages that follow [46].

## Research co-design and the role of context

Research co-design is defined as “the meaningful involvement of research users during the study planning phase of a research project” [1]. The planning phase refers to all activities that occur prior to finalizing a research question [1]. Co-design approaches may vary in terms of frequency and intensity, such as consultative (seeking the views of end-users), collaborative (establishing an on-going partnership between researchers and end-users) or end-user-led (shifting power to end-users who design and undertake the research and invite researchers to play a role) [1, 47].

Although research co-design can be used to enable end-users to contribute to study design and materials, in this paper, we focus specifically on the use of co-design for research prioritization and agenda-setting [1]. Research priority-setting has been defined as “a collective social activity for deciding which uncertainties are most worth trying to resolve through research” [48] and which are “most likely to improve service delivery and organization” [49]. Research priority-setting can occur at three levels [50]. At the macro level, the intent is to prioritize a broad topic area and a general direction for research. At the meso level, the intent is to prioritize a research question, which may be broad or narrow. At the micro level, the intent is to prioritize a focused research question. Common methods used for research prioritization include surveys, interviews, focus groups, consensus-building processes such as the Delphi or nominal group technique, and participation on advisory councils [1, 6, 51]. These methods may be deliberative, involving rating or ranking, fluid and open-ended via dialogue and debate, or a combination of both deliberative and fluid approaches [1].

The literature on research priority-setting acknowledges the importance of context. Authors argue that priority-setting methods should be tailored with consideration for contextual factors such as available resources, end-user interest and capability, and the nature of the sponsoring organization (e.g., academic, commercial, charitable, advocacy) [1, 51, 52]. They also speak to the geographical scope of priority-setting in terms of who is involved and who is the intended audience [52]. Research priority-setting can be broad in scope, conducted at a national or international level by pooling input from end-users spanning diverse contexts (e.g., [53–56]). While this approach generates research priorities that are more generalizable, it may not address the unique needs and challenges end-users face in *their* context. Other approaches to research priority-setting are narrower in geographic scope, focusing on a particular jurisdiction or locale (e.g., state/province, city, neighbourhood) [57, 58]. This approach reduces generalizability but enhances relevance and utility to immediate end-users.

We argue that healthcare PM demands a context-specific approach to research priority-setting in which the scope is limited to a particular PM system operating in a defined geographic, policy, time frame and service-delivery context. We make this assertion based on the state of the literature on healthcare PM. Studies on PM interventions in health systems report mixed results on their effectiveness as well as unintended negative consequences [34–45]. Furthermore, studies are often not designed or described in ways that capture the real-world complexities of PM, such as pertinent contextual factors (e.g., PM interventions that coexist with but are outside the scope of the study, data collection infrastructure, leadership, stakeholder perspectives). The mixed evidence base and shortcomings in research design and reporting make it challenging for policy-makers and leaders to extrapolate from the results to inform local decision-making and practice. We also may not be asking questions or designing research in ways that are meaningful to and actionable by end-users.

## Methods

This study was rooted in Dewey’s philosophical tradition of pragmatism, in which the focus is on people’s practices and experiences, rather than on abstract, and potentially constraining, theories about human nature [59]. For example, contrary to a structural model in which humans are “captive rule followers” or a rational actor model in which humans are “optimizers with well-defined preference ordering”, a pragmatic model stresses how humans experiment and learn in their context [60]. Pragmatism thus offers a richer and more realistic view of human behaviour. In his description of pragmatism, Dewey advocated for joint inquiry in which people jointly explore problems and solutions [59]. Research priority-setting with diverse types of end-users innately privileges people’s situated and lived experiences of a phenomenon and seeks to capture the importance of these experiences in the broader sociopolitical context in which they unfold.

### Study context

Located in Central Canada, Ontario is Canada’s most populous province with a population of 14.5 million [61]. Cancer Care Ontario (CCO), which houses the Ontario Renal Network (ORN), is a crown agency owned by the Government of Ontario but operates at arm’s length from the government. CCO is responsible for monitoring and improving cancer and renal care on behalf of the Government of Ontario. CCO is a “Network Administrative Organization” [62], a governance body that funds and oversees the performance of 13 regional cancer networks and 27 regional renal networks. The province is divided into geographic regions to organize care delivery; each network operates in a specific region, and together the networks cover the full geography of the province, which includes urban, rural and remote areas. Each network has a designated hub hospital. Each hub hospital’s cancer and/or renal programme oversees the performance of partner hospitals within their network boundaries. In other words, CCO primarily interfaces with the hub hospital programmes, who, in turn, interface with their partner hospitals and with front-line providers.

CCO provides the networks with performance standards/expectations and a robust and well-established PM system to assess, monitor and incent performance. The PM system consists of a variety of tools and processes: funding contracts stipulating performance expectations/deliverables with funding at risk of withdrawal for noncompliance; a regional scorecard with indicators, targets and network rankings; access to performance data through electronic platforms; quarterly performance review reports and meetings; annual performance recognition certificates; an escalation process for poor or declining performance; and public reporting of performance on select indicators [63–67]. CCO’s PM tools and processes are described in more detail in Additional file 1.

In 2016, CCO adopted an embedded researcher model to help establish a programme of research on PM. An embedded researcher is an individual whose main purpose is to carry out research through a collaborative process by working inside a host organization as a member of staff while also maintaining an affiliation with an academic institution [68]. The first author was thus hired as a staff scientist on an evaluation unit within the organization. In 2019, after the completion of this study, CCO was incorporated into a new agency, Ontario Health. CCO’s programmes and services remain unchanged.

### Data collection and analyses

The approach to research co-design was both collaborative and consultative [1]. As an embedded researcher inside CCO, the first author worked closely with CCO leaders, engaging them in regular discussions and decision-making around the PM research (collaborative approach). Through interviews, we also sought the views of a broader group of end-users involved with or influenced by PM (consultative approach). The co-design process consisted of five phases: (1) rapid review of the literature on PM, (2) informal discussions with CCO leaders about their information needs, (3) development of five potential research directions with CCO leaders based on inputs from steps 1 and 2, (4) end-user interviews and ranking of research directions and (5) prioritization and refinement of selected research direction with CCO leaders. We briefly describe all five steps below, but the focus of this paper is on the results of steps 4 and 5.

#### Steps 1 and 2: rapid review and informal discussions with CCO leaders

The first two steps—the rapid review and informal discussions with CCO leaders—occurred concurrently and iteratively such that the perspectives of CCO leaders shaped the review and the emerging results of the review informed further discussions with CCO leaders. On average, researchers met monthly with CCO leaders to plan and interpret the emerging results of the rapid review. A rapid review is “a form of knowledge synthesis in which components of the systematic review process are simplified or omitted to produce information in a timely manner” [69]. The primary intent of the rapid review was to identify gaps, debates and recommendations in the literature to inform the development of potential research directions. Searches were centred around the term “performance management” because it is often used as an umbrella term for different types of performance interventions (e.g., financial incentives, scorecards, rankings) and because it is the most commonly used term across different disciplines (e.g., business, healthcare, public management). We searched PubMed using the following search strings: (1) “performance management” AND (“healthcare” OR “health care” OR “health system”), (2) “performance management” AND (“policy” or “inter-organizational” OR “region*” OR “network”), and (3) “performance management system”. We supplemented this search by using the first string only to search the EMBASE database and by reviewing the reference lists of included papers to identify additional relevant papers. To be included, papers had to focus on or be relevant to PM at the interorganizational or network level. Papers on PM at the individual and team levels were excluded. Papers on PM at the organizational level were included if they imparted lessons applicable to the system level. Ultimately, 115 papers were included in the rapid review (see Additional file 2 for a PRISMA diagram summarizing the screening process).

The following data were systematically abstracted from the included papers: study aim, methods, definition/conceptualization of PM, PM components, PM barriers and enablers, study results, and gaps, debates or recommendations for future research and practice. To inform research priority-setting, we reviewed the abstracted content in the last category, namely the identified gaps, debates and recommendations (see Additional file 3 for a summary). In general, the rapid review pointed to two overarching research gaps. The first research gap was a need for in-depth studies that examine PM systems in their entirety (as opposed to examining specific PM interventions in isolation) and that provide rich descriptions of context and of multiple stakeholder views. Addressing this research gap seemed more conducive to qualitative methods. The second research gap was a need for more outcomes studies that determine the costs and effects of PM. Addressing this research gap seemed more conducive to quantitative methods. The two overarching research gaps were discussed with CCO leaders. The first research gap aligned well with the needs and practical concerns of CCO leaders. They emphasized the importance of hearing stakeholder experiences and views in their own words, and on the preference to examine the PM system holistically rather than focus on one or more specific components. They also argued that experimental research to test different PM approaches across the networks was not feasible due to limited resources to manage multiple PM approaches in an already complex and strained healthcare delivery system. Finally, they expressed concern about “survey fatigue” among stakeholders. Qualitative research methods involving dialogue with stakeholders about the PM system and a strong orientation to context was thus most appealing to CCO leaders.

#### Step 3: development of research directions

Based on the results of the rapid review, potential research directions were identified collaboratively by two researchers and six CCO leaders representing multiple hierarchical levels in the organization. The research directions were debated until consensus was reached using three criteria: relevance to CCO’s context and PM system, feasibility of the associated research methods, and potential to inform decision-making and practice. Research directions that failed to meet any one of these three criteria were removed by consensus. The final five research directions reflected both empirical gaps and practical, context-specific needs (Box 1). The wording of these research directions was jointly determined by the researchers and CCO leaders.

Box 1. Research directions on healthcare performance management

1. Examine how organizations/networks use CCO’s PM tools and processes and determine how to encourage and facilitate productive use of the PM system and its data
2. Describe and compare PM tools and processes across high-performing healthcare delivery systems (including CCO)
3. Explore how environmental and organizational factors influence compliance with CCO’s performance requirements and continuous improvement
4. Examine which of CCO’s PM tools and processes are most effective in motivating performance improvement
5. Identify unintended negative consequences stimulated by CCO’s PM system and determine how important they are and how they can be mitigated

**Step 4: end-user interviews and ranking of research directions**

After the list of five potential research directions was established, interviews with end-users commenced. The interviews served two purposes. First, we aimed to understand how administrative and clinical stakeholders, within CCO and across the networks it governs, perceive the role of CCO and the function and impact of its PM system (these results are presented in a separate paper). Second, we aimed to assess and prioritize research directions on PM with those involved with or affected by CCO’s PM system. This paper focuses exclusively on data related to the second aim.

To be eligible for an interview, individuals had to be employed by CCO or a regional network and involved in or affected by CCO’s PM system. A list of CCO representatives eligible for the study was generated by the PM Units of the cancer and renal branches of the organization. The list included all senior executives at CCO, all PM Unit managers and staff, as well as managers, clinical leaders and staff involved in or affected by PM from across all relevant departments and programmes within CCO. For recruitment of individuals within networks, an invitation was emailed to all Regional Vice Presidents (cancer) and all Regional Directors (renal). Networks that agreed to participate were asked to provide a list of staff and clinical leaders involved with or affected by CCO’s PM system. External representatives (not employed by CCO or the networks) were identified by CCO’s PM manager and the research team based on familiarity with CCO’s PM system and expertise in PM, including two patients, two researchers, one policy-maker and one representative from another provincial agency known as the Local Health Integration Networks (LHINs). The two patients had formerly sat on a PM committee at CCO and were actively involved in CCO’s Patient and Family Advisory Council. The purposeful sampling approach described above was supplemented by snowball sampling. At the end of each interview, the interviewer asked participants to nominate additional individuals for potential participation.

The first author conducted semi-structured individual and group interviews between 2017 and 2018 (see Additional file 4 for interview guide). The interviews lasted 1 hour, and approximately 20–30 minutes of that time was allotted to discussing PM research priorities. Patient interviews were conducted one-on-one. Other participants had the option of participating in a group interview or an individual interview. Group interviews were often preferred by participants for two reasons: (1) because they occur in a social context allowing participants to bounce ideas off one another and (2) because they are efficient and minimize ongoing disruption to programme operations. Individual interviews were offered as an option for those who preferred to speak with the lead researcher one-on-one, who could not attend their team/programme’s group interview or who were not part of a team/programme scheduling a group interview. Group interviews included immediate team/programme members only (i.e., a mix of administrative and clinical professionals from different hierarchical levels were included, but these individuals all worked together within the same team or programme). Group interviews ranged in size from two to nine individuals with an average of 3.5. Group interviews with network representatives were usually specific to one clinical area (cancer or renal); however, in some networks, a mix of representatives were included due to a shared leadership model (i.e., the Regional Vice President’s portfolio included both cancer and renal care). Interviews with CCO representatives were primarily conducted in person, while those with network representatives were primarily done by phone due to geographic dispersion.

Following a general conversation about CCO’s PM system (as per the first aim of the interviews described above), participants were asked an open-ended question about their “burning questions” regarding PM and what topics or issues they think would be most useful on which to conduct research. Following this open-ended, participant-led conversation, participants were given the list of five research directions. They were asked to “think out loud” as they read each option, providing their assessment based on three criteria: (1) Importance: does it address an important gap or need that is unlikely to be addressed through other means? (2) Impact: does it have strong potential to inform decision-making, change current practice, improve network performance or improve CCO’s performance? (3) Feasibility: will stakeholders be interested and willing to participate, and do we have the time and resources to execute the research? The use of criteria to focus the discussion and to balance competing dimensions is considered a best practice in priority-setting exercises [70].

Towards the end of the interview, participants were asked to rank order the five research directions from 1 (highest priority) to 5 (lowest priority) and to explain their ranking choices. We opted for a metric-based approach to priority-setting in which we aggregated individual rankings, rather than a consensus-based approach in which participants are exposed to the views of others and have an opportunity to reconsider their ranking [70]. In group interviews, participants were asked to write down their ranking independently and then share their ranking verbally with a brief explanation of their decisions. After ranking the research directions, participants were invited, for a second time, to offer alternative research directions. The interviews were digitally recorded and professionally transcribed verbatim. Ethical processes were followed in the collection and use of interview data, including making raw data only available to the first author, obtaining informed consent verbally at the beginning of each interview and guaranteeing participants’ confidentiality and anonymity in the reporting of study results.

**Step 5: prioritization and refinement of selected research direction**

The interviews transcripts were coded inductively and analysed thematically [71] to identify common patterns across participant responses in terms of suggested research directions and assessment of the proposed research directions. In the participant quotes provided below, we use the notation “P” for individual interviews and “G” for group interviews. In group interviews, it was not always possible to identify the role of the speaker (administrative versus clinical) or the clinical area they represent (cancer versus renal); therefore, some quotes indicate role and clinical area, while others do not.

The quantitative rankings were analysed using four methods: mean, median, frequency ranked first, and frequency ranked fifth. However, during analysis the research team noticed that the research direction most often ranked first was also very frequently ranked fifth, indicating strong divergence in preferences across stakeholders. Therefore, it was determined that frequency ranked first or second would be a more appropriate indicator of stakeholder consensus than frequency ranked first. Results for all ranking assessment methods are presented in Additional file 5. Results across the ranking assessment methods aligned, and we therefore only present the mean rankings in the paper.

As noted above, separate analyses were conducted on the quantitative and qualitative data. The two data sets were then integrated through a narrative approach [72] in which participants’ qualitative comments were used to contextualize and interpret their quantitative rankings. Based on these results, a research direction was selected, and minor modifications to scope and wording were applied by the research team in consultation with CCO leaders.

## Results

Seventy-three interviews (40 individual and 33 group) were conducted with 156 end-users representing a broad range of end-user groups, as shown in Table 1. Table 2 displays the mean ranking for each research direction overall and by end-user group. Table 3 presents positive and negative feedback for each of the five research directions which help explain the quantitative rankings.

**Table 1:** Participant demographics (*n* = 156)

Demographic characteristic	Percentage (%)
**CCO end-users**	
Role	
Administrative	85 (50/59)
Clinical (i.e., provincial clinical leads)	15 (9/59)
Department/portfolio	
Clinical Programmes and quality initiatives (cancer only)	36 (21/59)
Ontario Renal Network (renal only)	22 (13/59)
Prevention and cancer control (cancer only)	17 (10/59)
Planning and regional programmes (cancer only)	15 (9/59)
Corporate executives & corporate strategy (cancer and renal)	10 (6/59)
**Network end-users**	
Type of network	
Cancer	59 (52/88)
Renal	41 (36/88)
Role	
Administrative	74 (65/88)
Clinical (i.e., network clinical leads)	26 (23/88)
Cancer network performance ranking on scorecard (Fall 2017)^a^	
Low (ranked 11–14)	29 (4/14)
Medium (ranked 5–10)	43 (6/14)
High (ranked 1–4)	14 (2/14)
**External end-users**	
Patients	22 (2/9)
Policy-makers	11 (1/9)
Provincial agency leaders	44 (4/9)
Researchers	22 (2/9)

^a^Renal networks are not ranked on their overall performance; rather they are ranked on an indicator-by-indicator basis

**Table 2:** Rankings of performance management (PM) research directions overall and by end-user group

	Research directions (RDs)				
End-user group	RD1 Use of PM	RD2 PM in high-performing systems	RD3 Influence of context	RD4 Motivating improvement	RD5 Unintended negative consequences
All end-users (*n* = 156)	2.90	2.93	2.95	**2.68**	3.52
CCO cancer staff (*n* = 50)	**2.3**	3.0	3.7	2.6	3.5
CCO renal staff (*n* = 9)	3.2	**1.8**	3.1	2.6	4.3
Network cancer staff (*n* = 52)	3.3	3.0	**2.6**^a^	2.6	3.5
Network renal staff (*n* = 36)	3.1	3.2	**2.4**	2.9	3.2
External end-users (e.g., patients, policy-makers) (*n* = 9)	2.8	**2.1**	3.3	2.4	4.3

A lower number indicates a higher priority ranking. The highest priority research direction ranking per stakeholder group is in bold text

^a^Although there was a tie for highest mean ranking among network cancer staff, the median indicated a preference for RD3

**Table 3:** Sample stakeholder quotes for each research direction

Research direction	Positive feedback	Negative feedback
Examine how organizations/networks use CCO’s PM tools and processes, and determine how to encourage and facilitate productive use of the PM system and its data	“It would be good to figure out is there is a relationship between the performance of a region, and how they are using our system” (P04, CCO, cancer, manager) “It’s obvious to me that some regions apply it more effectively than others. There are some regions where their performance changes and there are ones which don’t. And there is something that they’re doing with what CCO gives them. Or maybe they’re not and they have some other things that they’re doing. Either way we should be learning from what they’re doing and seeing how CCO could be better supporting that, for them to make changes” (P07, CCO, cancer, manager)	“I think you’re going to get very different results for the 14 regions…it’s probably going to be a mishmash of things” (P17, CCO, cancer, administrative staff) “I think how they use the tools is just a symptom of other things…I think there’s a reason there are differences in the way the regions are using the tools, and it gets more into the local environment” (P18, CCO, cancer, administrative staff) “I think it’s a bit top-down and not stepping back enough. If there is an opportunity as a province to improve, you wouldn’t keep your head down and stay where you’re at. So, I’m not sure how much this is going to help us to move things forward” (G19, network, cancer and renal)
Describe and compare PM tools and processes across high-performing health systems, including CCO	“I feel like we should be learning from what other people do” (P07, CCO, cancer, manager) “I think we should reach out to those who performance manage well…Go there, learn the nuts and bolts of how they do it, bring that back, and assimilate the best practices that they use” (P57, CCO, cancer, clinician) “We think we’re doing a great job and we feel a sense of real ownership of how CCO measures performance. I would certainly love to know how that looks against other models” (G13, network, cancer) “See what the best are doing and steal shamelessly—Yes! It is hard for me to advise on the feasibility of implementing what is learned, but benchmarking against other jurisdictions is vitally important” (P16, patient)	“Do I ultimately believe we’ll get something revolutionary out of this? No. Their set-up is not the same as ours…Most of our tools are homemade tools which have evolved over the years. So, will their tools really fit here, into this environment? I don’t know. I would put it towards the bottom” (P46, CCO, cancer, manager) “The problem is defining what’s high-performing because there was a recent presentation that the UK has the highest performing health system out there, and yet they’ve got the worst cancer survival rates in the Western world” (G15, network, cancer) “It’s interesting, but I don’t know that it’s practical and applicable. Would it be of much help? Can you translate it to our context?” (G21, network, cancer and renal)
Explore how environmental and organizational factors influence compliance with CCO’s performance requirements and continuous improvement (e.g., competing accountability requirements, organizational culture, leadership style, CCO-network relationships)	“I think it would go a long way to helping educate CCO on what those factors are, so that we better understand the regions, and why they respond the way that they do. I think it will enable us to do a better job” (P18, CCO, cancer, administrative staff) “It’s important because we are ranked against one another even though we may have very different resources, demographics, and barriers that influence performance, so how that can be taken into consideration or supported from a provincial level? I think it’s very important to consider that when we’re looking at performance and outcomes. One size doesn’t fit all” (G3, network, cancer) “They will listen to you after the fact, if you’re not where you need to be, based on your local factors. But, I do think that needs to be taken into consideration up front, at the beginning of the process, rather than at the end” (G9, network, renal) “This one has applicability beyond the cancer programmes. The findings could be a very helpful tool to hospitals in Ontario” (G11, network, cancer and renal)	“I think it’s not useful, unless you have the capacity and ability to change the environmental and organizational factors” (P42, CCO, cancer, manager) “How will we be able to apply the results from this? I really do worry this will end up being too abstract and too far into the weeds of local issues…when you talk about leadership style, for example, what would you do with that information at a provincial level?” (G10, network, cancer and renal) “We sometimes get bogged down in conversations about how our unique characteristics might be a reason why we’re not performing and I think we need to avoid those types of conversations…the indicators are meant to have broader applicability…this is what we want to do everywhere for all patients no matter what door you’re walking through” (G21, network, cancer and renal)
Examine which of CCO’s PM tools and processes are most effective in motivating performance improvement	“I think this is really important, in part because I think we need more guidance on how to incent the change that we want” (P2, CCO, cancer, manager) “How we’re using them is not as important as why we’re using them. And you can get at the why by looking at what motivates a response and what does not” (G11, network, cancer and renal) “I really like this one. I mean at the end of the day what are we trying to do? We’re trying to change behaviour either that of the system or of individuals to improve, right? So, what methods instigate a change in behaviour or practice?” (G20, network, cancer and renal) “My largest interest is in this one as it relates to getting information down to the care providers and empowering a large roster of staff to buy-in or abide. That, in fact, might be my only interest” (G14, network, renal, clinician) “Metrics only work if the entity being measured feels compelled to engage and align to the metric” (P16, patient) “Some programmes really do seem to respond to these performance management initiatives that we have and others don’t, and, boy, would I ever like to know why because then you could make them more effective and you can reach your goals more easily” (G18, network, renal, clinician)	“I think that they work in combination with each other, so I am not sure…maybe it’s not which ones motivate improvement, but when do they, together as a toolbox, motivate improvement and when do they not and why?” (P07, CCO, cancer, manager) “I feel like if you ask that question you’re going to get very different answers from one programme to another” (P3, CCO, cancer, manager) “Some are going to be better for some centres and worse for others, and it’s just basically looking at what we’re doing now” (G12, network, renal) “I think your question is ambiguous because you’re looking at effectiveness in motivating improvement. That’s a very qualitative question. I would just say, ‘most effective in achieving improvement’” (P47, CCO, cancer, clinician)
Identify unintended negative consequences stimulated by CCO’s PM system, and determine how important they are and how they can be mitigated	“If there is something damaging in our processes, the longer we maintain those damaging processes the worse it is. So, with a bit of a risk lens on that, it comes up in my ranking” (P28, CCO, cancer, manager) “I love this one. I’ve seen really interesting examples of unintended consequences. We need to plug in some mitigation factors” (P14, CCO, cancer, manager) “I don’t know that we every really talk about the unintended negative consequences because we’re so focused on the positive outcome we want to see” (P63, CCO, renal, manager) “I think this is potentially an important question to look into as well. There is always a concern about focusing on one metric too much and therefore ignoring everything else and detracting from quality in other areas” (G20, network, cancer and renal) “I thought that was important because it’s your risk management process. You need to know what the downside is, so it has to be relatively high up in the ranking. Because you can’t go into all this and not be aware” (G17, external, LHINs)	“I think it’s important, but I’m not sure how impactful it might be. Even if we know that it exists, is there something we can do about it, without causing other unintended consequences?” (P18, CCO, cancer, administrative staff) “I am hesitant because it has a more negative spin on things. And, for me, it’s just trying to focus on what we can do better” (P07, CCO, cancer, manager) “I don’t think it will move us forward in terms of performance management, beyond doing some tweaks around some pain points” (P71, CCO, cancer, manager) “I think that unintended negative consequences don’t need research. There’s going to be somebody screaming really early on in the process. It doesn’t require research to summarize that” (G1, network, cancer) “It’s kind of a negative spin and I think it will come out in other ways, so I think that that one would be less of a priority to me” (P40, external, policy-maker)

End-user groups prioritized different research directions based on their experiences and information needs (Table 2). Internal CCO cancer staff were most interested in understanding how the networks use CCO’s PM tools and processes. They described anecdotal awareness that networks were leveraging performance data and associated tools differently. They thus wanted to better understand what high-performing regions were doing so that they could better support and spread those practices across networks.

Internal CCO renal staff and external representatives, including patients and managers from other provincial agencies, were most interested in learning from the PM approaches of high-performing healthcare delivery systems. They argued that “benchmarking” CCO’s PM system against others is an effective means to evaluate the system and to facilitate learning and improvement. It is important to note the small sample sizes for these two stakeholder groups (*n* = 9 each) and that they made up a small percentage of the overall sample of participants (18/156 or 11.5%). Nevertheless, in the overall sample, this research direction was most often ranked first, but also very frequently ranked fifth, indicating strong divergent reactions among participants (see Additional file 4). Critics of this research direction raised concerns regarding defining and identifying high-performing healthcare delivery systems and the feasibility of transferring findings from other jurisdictions to the Ontario context.

Network staff in both the cancer and renal sectors were most interested in the influence of contextual factors on PM and improvement. They emphasized that a “one-size-fits-all” approach to PM, particularly with regard to ranking networks against each other, fails to consider the unique organizational or regional challenges networks face, some of which they have limited control over. Participants argued that better understanding the influence of organizational and environmental factors on PM and improvement could result in tailored approaches to PM and/or tailored supports from CCO.

Despite variation in preferred topics across end-user groups, the research direction on which of CCO’s PM tools and processes motivate performance improvement had the highest overall mean ranking and was most often ranked first or second and least often ranked fifth. Participants were drawn to the term “motivate”, which they saw as getting to the core of PM, that is how to stimulate action to improve. Participants also argued that this was the only research direction that had an inherent focus on the influence of PM on front-line providers. They also felt this topic would generate insight into when, how and why PM works or fails to work.

The final research direction, on unintended negative consequences, was consistently ranked lowest by all end-user groups. Many participants had strong reactions to the idea of conducting research on a topic with a “negative spin on things”, and others argued that the results would only be of value if it was possible to mitigate identified unintended consequences without generating other unintended consequences.

## Potential research directions identified by end-users

End-users had an opportunity to suggest research directions on PM both before and after seeing the five research directions. Some research directions suggested by participants were already embedded in the existing five proposed by the research team. For example, numerous participants brought up competing accountability requirements as a barrier to PM, an issue that is captured under the research direction on organizational and environmental factors. Others suggested research directions that were deemed out of scope. For example, several participants identified indicator development and selection as an area ripe for additional research; however, the research team (and even participants themselves) noted that this was more within the realm of “performance measurement” than “performance management”.

The most common research suggestions from both CCO staff and network staff centred around four themes deemed within the scope of PM. The first theme was on better understanding the impact of PM on the experiences and behaviours of front-line providers. One participant asked, *“How do you get a data clerk and a front-line nurse to change their behaviour, how they interact with a patient, how do you get them to do that differently? Whether it’s picking a care setting or talking about goals of care, how do you change that behaviour?”* (P33, CCO, renal, manager). Another participant elaborated, *“If they [front-line providers] don’t believe in what they’re doing, they don’t do it…You can look at it at the system level, but it does come down to that individual motivation to create change”* (G16, network, cancer). A patient with cancer had a similar reflection on the need to focus on front-line providers: *“I think what will make any performance management system successful is if you understand your audience. You have to have a full and complete understanding of your front-line people”* (P20, patient). The topics of change management and clinician engagement were implicit in these sample quotes and explicitly raised by others. This theme highlights a need to understand how system-level PM trickles down to and influences front-line providers, if at all.

The second theme was on better understanding the impact of PM on patients and the patient experience, as this participant explained, *“I would like to know has the performance management approach truly affected change over other potential approaches? It’s all about the patient, it’s not about ticking the measurement box. Did the patient benefit?”* (P43, CCO, cancer, clinician). Another participant said, *“There is all this stuff just to meet the requirement as opposed to well why don’t we ask the doctors or nurses and patients in the clinic, has it made a difference in how patients are being cared for?…Sometimes we forget about that piece”* (G19, network, cancer and renal). Similarly, this participant said, *“Sometimes I feel if we just play the game we could be a perfect performer, but I’m not sure our patients would be any better off. We would just learn how to play the game of being a good performer”* (G15, network, cancer). This theme emphasizes the patient voice and challenges the notion that better performance as per an indicator or scorecard means better patient experience or patient outcomes.

The third theme was on pilot testing different approaches to PM across the networks and comparing outcomes. This participant provided an example, *“We need to test the best way to make changes. Like if we do pay for performance in this [network] and we roll it out in a different way in this [network], which way worked better? How is uptake? How is it sustained?”* (P14, CCO, cancer, manager). A related idea centred around the need to examine whether indicator type should dictate the approach to PM. One participant explained, *“A lot of our tools and processes for performance management were designed around certain types of indicators, like radiation quality indicators. When we get into things like patient experience indicators or other things that don’t have the same clinical evidence and are not easy to put a fence around or hold someone accountable to…do those types of indicators need different approaches? It’s not just which of our performance management tools and processes work well, but what do they work well for?”* (P07, CCO, cancer, manager). This theme focuses on technical questions regarding the design and implementation of PM interventions.

The fourth and final theme was regarding the cost-effectiveness of PM, as described by one participant, *“You’re spending millions of dollars monitoring these [networks] every year, are you seeing an effect?”* (P47, CCO, cancer, clinician). Another participant implored, *“It’s all about value for the money. That’s what everybody is looking for”* (G1, network, cancer). This theme emphasizes an economic perspective and consideration for the potential opportunity costs associated with the current investment of time and resources in PM.

The research team and CCO leaders determined that the first theme identified by end-users—on front-line provider behaviour change—was closely aligned with the research direction that had the highest mean ranking among end-users (Table 2)—on motivating change. Therefore, a more explicit behaviour change lens was integrated into the research direction, as described below. The three other research directions identified by end-users were noted for future consideration as part of a long-term programme of research on PM.

## Selected and refined research direction

Based on end-user rankings, research direction #4 was selected for study and further refined using end-users’ qualitative feedback. CCO leaders were involved in the refinement process, and the revised aim and associated methodology were shared on multiple occasions with internal CCO staff and network leaders who were invited to provide additional feedback.

The original research aim was to “examine which of CCO’s PM tools and processes are most effective in motivating performance improvement”. The revised aim is to “explain how and when CCO’s PM tools and processes promote and achieve improvement (or fail to)”. The revised aim differs from the original aim in three key ways. First, the wording in the original aim (“which…are most effective”) implied that the PM tools and processes would be examined separately as independent interventions rather than as interdependent components of a system. Second, the original aim did not acknowledge the role of context in shaping how and when PM works. Third, the original aim focused exclusively on motivation as the outcome of interest. Yet, behaviour change is the primary outcome of interest, and according to the COM-B model of behaviour [73], motivation is one of three necessary conditions for behaviour change; the others being capability and opportunity. The revised aim addresses these issues and gaps, resulting in a more robust research direction that aligns with end-user interests and needs and better responds to what we know and do not know from the literature.

## Discussion

Despite increasing interest in joint research priority-setting in both the healthcare and management disciplines, most published examples focus on improving clinical care and the patient experience [74] or on addressing the needs of marginalized groups [57], such as workers with disabilities [24] or the homeless [75]. Few research priority-setting exercises focus explicitly on healthcare management issues that occupy the space between policy and direct service delivery. Furthermore, many research priority-setting processes occur in a vacuum—devoid of context—perhaps driven by an intent to identify generalizable priorities. Finally, most studies focus on limited end-user groups, such as only patients and the public or only patients and clinicians. Few studies bring together diverse end-user groups to jointly set research priorities, including patients, clinicians, managers, policy-makers and researchers—and when they do, researchers often continue to dominate the sample [49]. Our research priority-setting study addressed these gaps in the literature. We focused squarely on a management issue (with policy implications)—that is, what do end-users want to know about managing the performance of healthcare delivery systems? We limited the scope of the study to a particular geographic and policy context: PM in cancer and renal care in the province of Ontario, Canada. We involved multiple end-user groups in research priority-setting with managers and clinicians dominating the sample as the two groups most involved with and affected by PM.

This study was undertaken by a researcher embedded in CCO, a government agency that uses a robust PM system to monitor and improve performance of 40 cancer and renal care networks. As a government agency, CCO is accountable to the public and serves an important knowledge transfer role between research and practice communities and with patients and the public. Hiring an embedded researcher and jointly setting research priorities with end-user groups in an unconventional topic area not directly related to clinical care serves the democratic ideals of accountability, transparency and responsiveness [76]. CCO’s approach and our experience serve as a model for other agencies that are committed to evidence-based decision-making.

At the outset of the research priority-setting process, we did not envision the involvement of over 150 end-users—an unusually large sample size for a qualitative study. The large sample size was driven, in part, by the broad range of relevant end-user groups and a desire to secure adequate representation from these groups. Furthermore, through word of mouth and snowball sampling, we found interest and enthusiasm among end-users to have critical conversations about PM. Rather than set an arbitrary limit on the sample size or cease data collection at the point of data saturation (i.e., when no new information is discovered), we continued to conduct interviews. We did this, in part, to achieve broad end-user engagement and ownership of the research agenda. We also realized that the priority-setting process was about more than the output of a research agenda. CCO leaders pointed out that the process itself was promoting a culture of engagement and improvement in the cancer and renal systems. Through the interviews, end-users were becoming more aware of their thoughts and feelings and their roles and interests related to PM [59]. In other words, the study was promoting learning and reflexive practice, often in a group context.

Our experience of the strengths and weaknesses of research co-design, including priority-setting, align with those highlighted in the literature [1]. We found that emerging results were immediately used by CCO. For example, results of the rapid review informed PM discussions and decisions about clarifying the primary function of CCO’s PM system and decreasing the volume of performance indicators monitored. It is also possible that joint research priority-setting improved participation in the study. Combining a literature review, open-ended dialogue via interviews, and a deliberative ranking method enabled us to leverage existing evidence and the strengths of both qualitative and quantitative data. This multi-method approach is common in established research priority-setting methods including the JLA method [14, 77]. Finally, in terms of potential weaknesses, the co-design process took a considerable amount of time and resources to execute. CCO leaders saw this as a worthwhile investment in a long-term programme of research on PM. However, the research process overall—including coding and data analysis, writing and publishing scholarly manuscripts and securing grant funding—all took longer than expected due both to limited resources and the rigor applied throughout the process. The risk of identifying research priorities jointly with end-users is that by the time the selected research is executed, the context may have shifted, new issues may have arisen, and the applicability of the results may be in question.

A pertinent example of a “shifting context” for our study was the emergence of the global COVID-19 pandemic in 2020. The pandemic shifted operational priorities for cancer and renal networks (e.g., managing backlogs and coping with patients presenting at a later disease stage). It is unclear whether research priorities on PM have shifted because of the pandemic. It is possible, for example, that participants would ascribe more importance to the study of unintended negative consequences of PM because of the pandemic. However, it is more likely that research priorities on performance *measurement* have shifted, fuelled by a desire to measure less due to competing priorities and to measure different things to align with new needs and challenges associated with the pandemic.

With a jointly identified and refined research direction in hand, what are the next steps for us? To examine how and when CCO’s PM tools and processes promote and achieve improvement, we are conducting retrospective comparative case studies of “most improved” and “least improved” networks on four CCO performance indicators. We selected one “most improved” and one “least improved” network per indicator, for a total of eight networks. Networks are matched on key structural characteristics (e.g., patient volumes, geographic location) to facilitate comparison. By examining most and least improved networks operating under the same PM system, we are examining how, when and why PM stimulates (or fails to stimulate) local action to improve. In line with the study’s focus on behaviour change, a key feature of this study is the participation of front-line providers, not just administrative and clinical leaders. All eight of the networks we purposefully selected to participate in the study accepted the invitation and dedicated resources to gathering relevant materials for document review, facilitating a site visit and enabling leaders and staff to participate in interviews. Their interest and engagement in the study may reflect the perceived applicability and credibility of the research given the joint priority-setting process.

The concept of behaviour change is not new to the PM field. The inherent aim of PM is to change behaviour [33]. Likewise, PM is not new to the behaviour change field. Behaviour change techniques include goal-setting, performance feedback, comparison of performance, rewards and punishments [78]—all of which are PM strategies as well [33]. Therefore, the two concepts are inherently intertwined. Yet, most studies of PM use organizational theories, most notably agency and stewardship theories, or draw from a limited range of behaviour change theories rooted in psychology, such as contextual feedback intervention theory, expectancy theory and goal-setting theory [45, 79, 80]. Often, these studies focus on PM at the individual, team or organizational level, not the network level. Few studies of PM consider behaviour change theories explicitly and comprehensively, for example, by using the behaviour change technique (v1) taxonomy (BCT) [78] and theoretical domains framework (TDF) [81]. The BCT and TDF consolidate over 30 behaviour change theories into comprehensive taxonomies of behaviour change techniques, mechanisms and influencing factors. To our knowledge, the BCT and TDF have not been applied to healthcare PM. This may be due to the breadth of content in the frameworks and the challenges associated with linking individual behaviour change with network-level phenomena. Our comparative case studies will leverage the BCT and TDF to examine the role and influence of CCO’s PM system at multiple levels of analysis: network, organization, programme and individual.

### Limitations

This study has limitations. First, studies of PM in healthcare are fragmented across diverse disciplines, including the health sciences, public management and business. As such, our rapid review of the literature to inform the development of research directions may have missed relevant literature. Furthermore, we did not assess the quality of studies included in the review. However, it was not our intent to conduct a comprehensive or systematic review, and it is common for rapid reviews to limit sources and to omit stages of traditional reviews such as quality assessment [69].

Second, the priority-setting process was largely driven by the five research directions identified and refined by the research team and CCO leaders. There are two potential limitations related to this. First, the research directions did not emerge from interview participants themselves. However, participants were asked for their ideas regarding potential research directions both before and after being presented with the five research directions. Furthermore, we would argue that combining a rapid review with end-user views ensures both academic and practical perspectives are incorporated into decision-making and could be considered a strength of the research design. The second potential issue regarding the five research directions is their broad scope and wording. The priority-setting process may have generated different results with precise research questions rather than broad research directions. For example, we realized during the priority-setting process that the five research directions were not necessarily mutually exclusive. For example, some participants argued that an analysis of contextual factors (research direction #3) could be incorporated into any of the other research directions. Indeed, this is what we chose to do when refining the selected research direction (research direction #4) and designing a study to address it. Different wording may have also generated different rankings. For example, the fifth research direction on unintended negative consequences may have been ranked differently if framed in terms of unintended negative *and* positive consequences of PM.

Third, the sample, while large, was not equally representative across end-user groups. Only nine “external representatives” (not employees of CCO or the networks) were included in the study (5.7% of sample). Furthermore, although almost a quarter of the sample consisted of practising clinicians (20.5%), these individuals were clinical *leaders* involved in CCO programmes and activities. We did not seek the views of front-line providers such as physicians not in leadership roles, nurses and allied health professionals. Nevertheless, the importance of designing and evaluating PM systems with front-line providers in mind emerged as a key theme in the data and was incorporated into the selected research direction.

Fourth, we did not evaluate the priority-setting exercise itself by asking end-users about their experiences with the process or the outcomes. Such an evaluation, while important, would have required additional time and resources. Furthermore, given the high volume of surveys, interviews and less structured forms of consultation with stakeholders by CCO, there was concern regarding research fatigue.

Finally, the priority-setting process was specific to CCO’s PM system. Therefore, the results may not be generalizable. However, the research directions were informed by a rapid review of the literature and are broad in scope, suggesting that the results may be transferable to similar agencies’ PM systems and contexts.

## Conclusions

This study provides insight into what end-users want to know about managing the performance of healthcare delivery systems. The results suggest that end-users are keen to open the “black box” of PM to explore more nuanced questions beyond “does PM work?” End-users want to know *how*, *when* and *why* PM contributes to behaviour change among front-line care providers. In addition to identifying a high-priority research direction on healthcare PM that may be transferable to other contexts, the results of this study also raise practical implications for policy-makers and managers.

First, a “behaviour change” lens should be integrated into the design and implementation of PM systems. PM (re)design should involve formal analysis of the nature of the target behaviour, what conditions need to be altered to achieve behaviour change (these may be internal to individuals, or aspects of their social and physical environment) and the PM (or alternative) interventions required to change those conditions [73].

Second, during PM (re)design, explicit consideration should be given to whether a “one-size-fits-all” approach to PM is appropriate. PM could be tailored based on the context of an organization or network (e.g., custom, rather than uniform, performance targets), and/or tailored supports could be provided to facilitate organizations and networks in responding to PM (e.g., training in rapid cycle testing for quality improvement). PM could also be tailored to the type or nature of performance indicators. However, decisions regarding tailoring PM must be balanced by attention to equity in patient access to high-quality care regardless of location.

Third, and related to the above two recommendations, process evaluation methodology should be embedded in all assessments of PM interventions. Process evaluations focus on three key questions: (1) what was implemented and how, (2) how does the intervention produce change, and (3) how does context affect implementation and outcomes? [82] A process evaluation uses mixed methods to explain why an intervention did or did not produce its intended effect. The results of a process evaluation can be used to inform corrective measures and the design of future interventions.

Fourth, mechanisms for supporting knowledge sharing and learning about PM across diverse health systems are needed. Traditional academic papers often fail to provide the context and detail that policy-makers and managers need to inform local decision-making and practice. Practitioner end-users want to know the operational day-to-day details of how PM is executed and how it evolves (or not) over time—and why, that is, what contextual factors are at play? When executing research on PM, researchers should comprehensively describe the PM intervention(s) under study and the context as this information helps end-users determine the relevance of study results to them. Other mechanisms for knowledge sharing may include publicly available reports, infographics on PM systems and operations [83], and learning collaboratives [84].

Fifth, the ultimate intent of PM—to ensure high-quality patient care—should remain the focus of PM. This can be achieved by selecting performance indicators that reflect the outcomes most important to patients and then aligning PM tools and processes to those outcomes. Additional strategies include involving patients in the design and evaluation of PM systems and ensuring patient-level data and patient stories are collected, analysed, reported and acted on as part of PM evaluation efforts.

In terms of future research, this study should be replicated in other contexts to broaden our understanding of end-user experiences and information needs regarding healthcare PM. Replication is particularly important given that our study was conducted before the COVID-19 pandemic. Those undertaking research co-design on healthcare PM should (1) include patients and their families, (2) evaluate the co-design process itself and (3) ensure adequate resources are in place to support rapid execution and dissemination of results without sacrificing research rigor.

Our rapid review and interviews also generated several potential research directions in need of further examination, particularly on *how*, *when* and *why* PM contributes to behaviour change among front-line care providers. Our results also emphasize the need to embed process evaluation into future research on PM interventions. As researchers work more closely with end-users, we can collectively build a body of knowledge that will contribute to “practice theory” regarding how PM is enacted day to day and why PM works or fails [85].

## Supplementary Information

**Additional file 1. **Cancer Care Ontario (CCO) performance management interventions organized by primary function.

**Additional file 2. **PRISMA flow diagram for rapid review on health system performance management.

**Additional file 3. **Rapid review of health system performance management: gaps, debates and recommendations for future research.

**Additional file 4. **Excerpt from interview guide.

**Additional file 5. **Research direction ranking analysis (*n* = 156).

## Data Availability

The data analysed during the current study is available from the corresponding author on reasonable request.
