# Supplementary material for: What do end-users want to know about managing the performance of healthcare delivery systems? Co-designing a context-specific and practice-relevant research agenda
Source: Health Res Policy Syst. 2021 Oct 11;19:131. doi: 10.1186/s12961-021-00779-x (PMC8504563; doi:10.1186/s12961-021-00779-x)
Supplement: Supplementary file 1 — Additional file 1. Cancer Care Ontario (CCO) performance management interventions organized by primary function. [file 12961_2021_779_MOESM1_ESM.docx]

**Additional File 1. Cancer Care Ontario (CCO) Performance Management Interventions Organized by Primary Function***

| **PM Intervention** | **Description** |
| --- | --- |
| Guiding |  |
| Network administrative and clinical leadership model | Administrative leaders are embedded in host hospitals and are employees of, and therefore accountable to, both their host hospital and CCO. Each clinical program within CCO has a ‘clinical lead’ counterpart within each network. Clinical leads are paid by CCO for one day per week. |
| Funding contracts | Specify expectations for patient volumes, data submission, and implementation of initiatives. Some funds may be withdrawn for non-compliance with these performance expectations/deliverables. |
| Monitoring |  |
| Scorecard | Generated quarterly to grade performance for each network (green, yellow, red) relative to each indicator and its target, and to rank performance of each network relative to others (global ranking in cancer for overall performance versus ranking per indicator in renal) |
| Annual Monitoring Report | Generated annually to monitor indicators retired from the scorecard (cancer only). |
| Web-based access to performance data | Secure, web-based database and analytic tools offer historic, current, and projected data on indicators; updated monthly and allows users to generate reports on specific queries. |
| Public reporting | The Cancer Quality Council of Ontario generates the Cancer System Quality Index, which is a web-based public reporting tool on cancer system performance across 30 indicators. Select renal indicators are also reported on the Ontario Renal Network website. |
| Improving |  |
| Quarterly performance review reports | Issued quarterly in preparation for quarterly meetings (below) and includes scorecard indicators as well as additional indicators; if performance is below target or declining for any given indicator, requires commentary on contributing factors and improvement plans; space also provided to summarize successes |
| Quarterly performance review meetings | Held between CCO leaders and network leaders following each quarter. Occurs via video-conference in cancer system and tele-conference in renal system. Performance results for the past quarter are discussed as well as data quality or reporting problems, success stories, challenges, and plans for improvement. |
| Recognition certificates | Issued annually for each indicator for networks that met target, were the top performer, and/or were the most improved. |
| Escalation process for poor or declining performance | Commences with an informal conversation with network leadership regarding performance, and may progress to a formal letter with template to complete a required improvement action plan and, in rare cases, to withdrawing of funds associated with requirement, if relevant. |

*Tripartite framework of PM functions adapted from [33]
