# Supplementary material for: What do end-users want to know about managing the performance of healthcare delivery systems? Co-designing a context-specific and practice-relevant research agenda
Source: Health Res Policy Syst. 2021 Oct 11;19:131. doi: 10.1186/s12961-021-00779-x (PMC8504563; doi:10.1186/s12961-021-00779-x)
Supplement: Supplementary file 2 — Additional file 2. PRISMA flow diagram for rapid review on health system performance management. [file 12961_2021_779_MOESM2_ESM.docx]

**Additional File 2. PRISMA Flow Diagram for Rapid Review on Health System Performance Management**

Records identified through database searching
(n = 388)

## Identification

Records after duplicates removed
(n = 255)

Records excluded
(n = 99)

Records screened
(n = 255)

## Screening

Full-text articles excluded
(n = 41)

Full-text articles assessed for eligibility
(n = 156)

## Eligibility

## Included

Studies included in rapid review
(n = 115)
