# Supplementary material for: What do end-users want to know about managing the performance of healthcare delivery systems? Co-designing a context-specific and practice-relevant research agenda
Source: Health Res Policy Syst. 2021 Oct 11;19:131. doi: 10.1186/s12961-021-00779-x (PMC8504563; doi:10.1186/s12961-021-00779-x)
Supplement: Supplementary file 3 — Additional file 3. Rapid review of health system performance management: gaps, debates and recommendations for future research. [file 12961_2021_779_MOESM3_ESM.docx]

**Additional File 3. Rapid Review of Health System Performance Management: Gaps, Debates, and Recommendations for Future Research**

| **Gaps** | **Debates** | **Recommendations for Future Research** |
| --- | --- | --- |
| Limited attention to network/system-level PM compared with PM at the individual, team, and organizational levels  Limited attention to PM compared with performance measurement (e.g., indicator selection)  Focus on a narrow selection of PM interventions, primarily public reporting and financial incentives  PM interventions often studied in isolation, even though in practice they co-exist  Inadequate descriptions of PM intervention(s) under study  Inadequate descriptions of context  Inadequate attention to implementation and uptake of PM; focus tends to be on strategic level issues  Lack of attention to evolution of PM systems over time  Inadequate attention to preferences, motivations, and experiences of healthcare professionals | What is the core objective of PM – accountability and/or learning? Should the objective influence PM design? Can accountability and learning co-exist as dual objectives of PM?  How are PM systems enacted and experienced?  Why is the evidence base on the effectiveness of PM so mixed?  How can unintended negative consequences of PM be mitigated? Are there unintended positive consequences of PM? | Focus on network/system-level inter-organizational PM  Go beyond performance measurement to examine influence of performance management strategies such as rewards and sanctions  Study PM systems in their entirety, including all relevant components  Conduct longitudinal research of PM  Conduct in-depth qualitative research or mixed methods research on PM  Examine diverse stakeholder experiences and perceptions of PM |
