# Supplementary material for: What do end-users want to know about managing the performance of healthcare delivery systems? Co-designing a context-specific and practice-relevant research agenda
Source: Health Res Policy Syst. 2021 Oct 11;19:131. doi: 10.1186/s12961-021-00779-x (PMC8504563; doi:10.1186/s12961-021-00779-x)
Supplement: Supplementary file 4 — Additional file 4. Excerpt from interview guide. [file 12961_2021_779_MOESM4_ESM.docx]

**Additional File 4. Excerpt from Interview Guide** (used for end-users in networks)

**Part 1: General Experiences and Views of System Performance Management**

1. What is your role in your organization, and to what extent are you directly involved in performance management activities?

…

1. Do you have any ‘burning questions’ regarding performance management?
   - Are there specific topics or issues that you think would be useful to conduct research on?

**Part 2: Research Prioritization**

In this part of the interview, I will show you a list of research directions that we developed based on the academic literature and informal discussions with CCO leaders. Please take a moment to read through all 5 directions. Please ‘think out loud’ as you review each one and feel free to ask any questions.

[Participant assessed each research direction and interviewer asked probing questions]

1. Would you like to add other topics to the list of research directions?
2. Now I would like you to rank the research directions in order of priority. To help you with your ranking, we have a list of three criteria you may wish to consider:
3. **Importance**: does it address an important gap or need that is unlikely to be addressed through other means?
4. **Impact**: does it have strong potential to inform decision-making, change current practice, improve regional performance, or improve performance of CCO?
5. **Feasibility**: will stakeholders be interested and willing to participate, and do we have the time and resources to execute the research?

With consideration for these three criteria, please rank the research directions from 1 to 5 with “1” indicating the highest priority and “5” indicating the lowest priority. Please ‘think out loud’ as you rank the research directions and feel free to ask any questions.
