# Supplementary material for: What do end-users want to know about managing the performance of healthcare delivery systems? Co-designing a context-specific and practice-relevant research agenda
Source: Health Res Policy Syst. 2021 Oct 11;19:131. doi: 10.1186/s12961-021-00779-x (PMC8504563; doi:10.1186/s12961-021-00779-x)
Supplement: Supplementary file 5 — Additional file 5. Research direction ranking analysis (n = 156). [file 12961_2021_779_MOESM5_ESM.docx]

**Additional File 5. Research Direction Ranking Analysis (n=156)**

The table below illustrates a preference for Research Direction (RD) #4 based on the mean, frequency ranked 1^st^ or 2^nd^, and frequency ranked 5^th^. Examining frequency ranked 1^st^ shows a preference for RD #2 and #3; yet, these research directions were also most frequently ranked 5^th^, demonstrating strong divergences in preference across end-users.

|  | **Research Directions** | | | | |
| --- | --- | --- | --- | --- | --- |
| **Analysis Method** | **RD1**  Use of PM | **RD2**  PM in High-Performing Systems | **RD3**  Influence of Context | **RD4** Motivating Improvement | **RD5**  Unintended Negative Consequences |
| Mean | 2.90 | 2.93 | 2.95 | 2.68 | 3.52 |
| Median | 3 | 3 | 3 | 3 | 4 |
| Frequency Ranked 1st | 32 | 42 | 41 | 33 | 8 |
| Frequency Ranked 1^st^ or 2^nd^ | 68 | 67 | 71 | 71 | 31 |
| Frequency Ranked 5^th^ | 30 | 36 | 37 | 17 | 36 |
